# Proteomic analysis of bone marrow-derived mesenchymal stem cell extracellular vesicles from healthy donors: implications for proliferation, angiogenesis, Wnt signaling, and the basement membrane

**DOI:** 10.1186/s13287-021-02405-7

**Published:** 2021-06-05

**Authors:** Jeffrey D. McBride, Luis Rodriguez-Menocal, Wellington Guzman, Aisha Khan, Ciara Myer, Xiaochen Liu, Sanjoy K. Bhattacharya, Evangelos V. Badiavas

**Affiliations:** 1 Phillip Frost Department of Dermatology and Cutaneous Surgery, University of Miami Miller School of Medicine, 1600 NW 10th Ave, RBSB 2023A, Miami, FL 33136 USA; 2 Interdisciplinary Stem Cell Institute, Miami, 33136 Florida (FL) USA; 3 Bascom Palmer Eye Institute, Miami, 33136 Florida (FL) USA; 4 Miami Integrative Metabolomics Research Center, Miami, 33136 Florida (FL) USA; 5 Katz Family Drug Discovery Center, Miami, FL 33136 USA

**Keywords:** Stem cells, Extracellular vesicles, Cell cycle, Angiogenesis, Wnt signaling, Basement membrane, Collagen

## Abstract

**Background:**

Bone marrow-derived mesenchymal stem cells (BM-MSCs) have shown therapeutic potential in various in vitro and in vivo studies in cutaneous wound healing. Furthermore, there are ubiquitous studies highlighting the pro-regenerative effects of BM-MSC extracellular vesicles (BM-MSC EVs). The similarities and differences in BM-MSC EV cargo among potential healthy donors are not well understood. Variation in EV protein cargo is important to understand, as it may be useful in identifying potential therapeutic applications in clinical trials. We hypothesized that the donors would share both important similarities and differences in cargo relating to cell proliferation, angiogenesis, Wnt signaling, and basement membrane formation—processes shown to be critical for effective cutaneous wound healing.

**Methods:**

We harvested BM-MSC EVs from four healthy human donors who underwent strict screening for whole bone marrow donation and further Good Manufacturing Practices-grade cell culture expansion for candidate usage in clinical trials. BM-MSC EV protein cargo was determined via mass spectrometry and Proteome Discoverer software. Corresponding proteomic networks were analyzed via the UniProt Consortium and STRING consortium databases.

**Results:**

More than 3000 proteins were identified in each of the donors, sharing > 600 proteins among all donors. Despite inter-donor variation in protein identities, there were striking similarities in numbers of proteins per biological functional category. In terms of biologic function, the proteins were most associated with transport of ions and proteins, transcription, and the cell cycle, relating to cell proliferation. The donors shared essential cargo relating to angiogenesis, Wnt signaling, and basement membrane formation—essential processes in modulating cutaneous wound repair.

**Conclusions:**

Healthy donors of BM-MSC EVs contain important similarities and differences among protein cargo that may play important roles in their pro-regenerative functions. Further studies are needed to correlate proteomic signatures to functional outcomes in cutaneous repair.

## Background

The relationship between the skin and other body tissues, such as the bone marrow, is complex and relies on the interaction and exchange of information and signals, including secreted proteins. The bone marrow appears to serve key roles in maintaining skin homeostasis. The relationship of the bone marrow to the skin is intricately connected via its secretome—the totality of proteins produced by the bone marrow that can serve functions in skin tissues. In patients that have dysfunctional bone marrow, the skin may be the first sign of an underlying pathology, through, for example, development of chronic wounds [1], changes in pigmentation, and infection. In subjects with genetic mutations resulting in dermatologic phenotypes, such as forms of epidermolysis bullosa, bone marrow transplants have been shown to be effective in attenuating skin pathology [2]. While bone marrow-derived mesenchymal cells (BM-MSCs) have been shown to be beneficial in a variety of diseases, including wound healing [3–5], but engraftment and survival into other tissues after transplant is very low, the exact mechanisms as to how patients experience benefit from cellular therapy remain to be fully understood. We hypothesized that the secretome of the bone marrow cells contains proteins important in skin structure (ex. basement membrane components) and function that may help explain, in part, the beneficial effects of bone marrow transplants and BM-MSC treatment in patients with cutaneous disease. In this study, using mass spectrometry, we analyzed the proteins in the secretome that co-purified with extracellular vesicles secreted by BM-MSCs from 4 healthy donors.

## Methods

### Bone marrow donors

Collection of primary human donor bone marrow was under the approval of the University of Miami Institutional Review Board (IRB) and in accordance with policies of the Interdisciplinary Stem Cell Institute. All experiments were performed in accordance with relevant guidelines and regulations and complied with the Declaration of Helsinki. Informed consent was obtained for all human subjects, and permission was given by all 4 human subjects to publish results derived from the tissues and cells and, if necessary, to publish any identifying information, including images. The human donors of the bone marrow were a 33-year-old male (donor 1), 33-year-old female (donor 2), 28-year-old female (donor 3), and 28-year-old male (donor 4). As is standard for bone marrow donors at the Interdisciplinary Stem Cell Institute, all 4 donors tested negative for anti-human immunodeficiency virus (HIV)-1/HIV-2, anti-human lymphotrophic virus (HTLV) I/II, anti-hepatitis C virus (HCV), HIV-1 nucleic acid test, HCV nucleic acid test, hepatitis B surface antigen (HBsAg), anti-HBc (core antigen) (IgG and IgM), anti-cytomegalovirus (CMV), West Nile virus (WNV) nucleic acid, *T. cruzi* ELISA (Chagas disease), rapid plasma regain (RPR) for syphilis, and had no clinical/history/laboratory evidence to suggest Creutzfeldt-Jakob disease. The bone marrow (approximately 80 mL) was aspirated from the posterior iliac crests as per standard practice of the University of Miami Bone Marrow (BM) Transplant Programs. The marrow was aspirated into heparinized syringes, and labeled syringes were transported at room temperature to the Good Manufacturing Practices (GMP) facility at the Interdisciplinary Stem Cell Institute at the University of Miami. BM was processed using Lymphocyte Separation Medium (LSM; specific gravity 1.077) to prepare the density-enriched, mononuclear cells (MNCs). Cells were diluted with Plasmalyte A or phosphate-buffered saline (PBS) buffer and layered onto LSM using conical tubes to isolate MNCs following established standardized operating procedures. The MNCs were washed with Plasmalyte A or PBS buffer containing 1% human serum albumin (HSA). The washed cells were sampled to determine the total number of viable nucleated cells. MSCs were initially cultured in Alpha-MEM media (Corning Cat. No 15-012-CV) supplemented with 2mM L-glutamine, 20% fetal bovine serum (FBS), 100 units/ml penicillin, and 100 μg/ml streptomycin. The expansion was performed in T175 cm^2^ flasks (Corning Cat. No 431466) at 37°C, using a 5% CO_2_ humidified incubator. MSCs were detached from the culture vessels using trypsin exposure, passaged, and cryopreserved at passage three prior to use in the following experiments. MSCs were verified in the GMP as viable, CD105^+^, CD45^–^ cells, sterile, mycoplasma-free, and endotoxin-free. Our previous work with MSCs of this nature revealed expression of HLA-class 1, CD90, CD73, and CD105 while being negative for CD45, and contained differentiation capacity into different lineages [6, 7].

### Isolation of EVs

Passage three cells were taken from cryopreservation, recovered, and cultured in T75 cm^2^ flasks (Corning Cat. No 3276) until 80% confluency, at which time the MSCs were washed several times with PBS, switched to serum-free Alpha-MEM media for 24 h to allow for EV collection into the serum-free media, which was then isolated and processed for downstream isolation using ExoQuick-TC® ULTRA EV Isolation Kit for Tissue Culture Media (Cat # EQULTRA-20TC-1), according to the manufacturer’s instructions. Dot blot was performed to verify extracellular vesicles were isolated without cellular contaminants (Exo-Check Exosome Antibody Arrays, Cat # EXORAY200A-4, Cat #EXORAY210A-8) according to the manufacturer’s instructions.

### Processing of EV samples prior to mass spectrometry analysis

Lysing the EVs were as follows (all reagents from Sigma, unless otherwise stated). Isolated extracellular vesicles were centrifuged for 10 min at 2000×*g* at 4°C. Samples were speed vacuumed dry until the sample was dry. Fifty microliters of 20 mM Tris-2% (sodium dodecyl sulfate) SDS was added. The mixture was heated at 95°C for 30 s and chilled for 30 s; this was cycled for a total of 5 min. Samples were sonicated for 1 min. Proteins were precipitated with cold acetone. Samples were speed vacuumed until dry and resuspended in 100 μL ammonium bicarbonate. Eight micrograms of protein was added, centrifuged for 10 min, and speed vacuumed until the sample was dry. Eight microliters of 50 mM ammonium bicarbonate (pH 7.8) was added to the samples. Samples underwent denaturation with 15 μL of 10 M urea in 50 mM ammonium bicarbonate (pH 7.8). Samples were reduced using 2 μL of 125 dithiothreitol DTT in 50 mM ammonium bicarbonate (pH 7.8). Samples were incubated for 1 h at room temperature. Samples underwent alkylation with 5 μl of 90 mM iodoacetamide in 50 mM ammonium bicarbonate, pH (7.8) and incubated in room temperature for 30 min. Samples were quenched with 3.33 μL of 125 mM DTT in 50 mM ammonium bicarbonate (pH 7.8). Samples were incubated at room temperature for 1 h in the dark. Ammonium bicarbonate (50 mM) was added to dilute urea to 1 molar concentration. Samples were digested with trypsin corresponding to 1:30 w/w enzyme to protein and incubated overnight at 37° for 18 h. Formic acid (50%) was added to stop trypsin reaction (5:100 v/v formic acid to sample). Samples were desalted using the Pierce C18 Spin Tips (Thermo Scientific). Trifluoroacetic acid (TFA) (2.5%) was added to the sample to adjust TFA concentration to 0.05%; pH of less than 4 was verified. C18 Spin Tips were used were placed into a spin adapter and the tip was wetted with 0.1% TFA in 80% acetonitrile (ACN) and centrifuged for 1 min. After discarding the flow through, the sample was added to C18 spin tip and centrifuged at 1000×*g* for 1 min; this process was repeated until all sample was passed through the C18 Spin Tip. The Spin Tip was then transferred to a fresh microcentrifuge tube. The sample was eluted by adding 20 μL of 0.1% TFA in 80% ACN and centrifuge at 1000×*g* for 1 min; this step was repeated again to further elute the sample. The sample was speed vacuumed to dry. The samples were reconstituted in 50 μL of 2% acetonitrile in LC-MS grade water with 0.1% formic acid prior to LC-MS/MS analysis.

### High-performance liquid chromatography (HPLC) and mass spectrometry

The following methods were performed as previously described [8]. In brief, reversed-phase chromatographic separation utilized an Easy-nLC 1000 system (Thermo) with an Acclaim PepMap RSLC 75 μm × 15 cm, nanoViper column (Thermo). The solvents were LC-MS grade water and acetonitrile with 0.1% formic acid. Peptides were analyzed using a Q Exactive mass spectrometer (Thermo) with a heated electrospray ionization source (HESI) operating in positive ion mode. Protein identifications from MS/MS data utilized the Proteome Discoverer 2.2 software (Thermo Fisher Scientific) using Sequest HT search engines. The data was searched against the *Homo sapiens* entries in Uniprot protein sequence database. The search parameters included precursor mass tolerance 10 ppm and 0.02 Da for fragments, 2 missed trypsin cleavages, oxidation (Met) and acetylation (protein N-term) as variable modifications, and carbamidomethylation (Cys) as a static modification. Percolator PSM validation was used with the following parameters: strict false discover rate (FDR) of 0.01, relaxed FDR of 0.1, maximum ΔCn of 0.05, and validation based on q-value. We obtained the high confidence peptides and filtered out the low and medium confidence peptides.

## Results

**Fig. 1:**
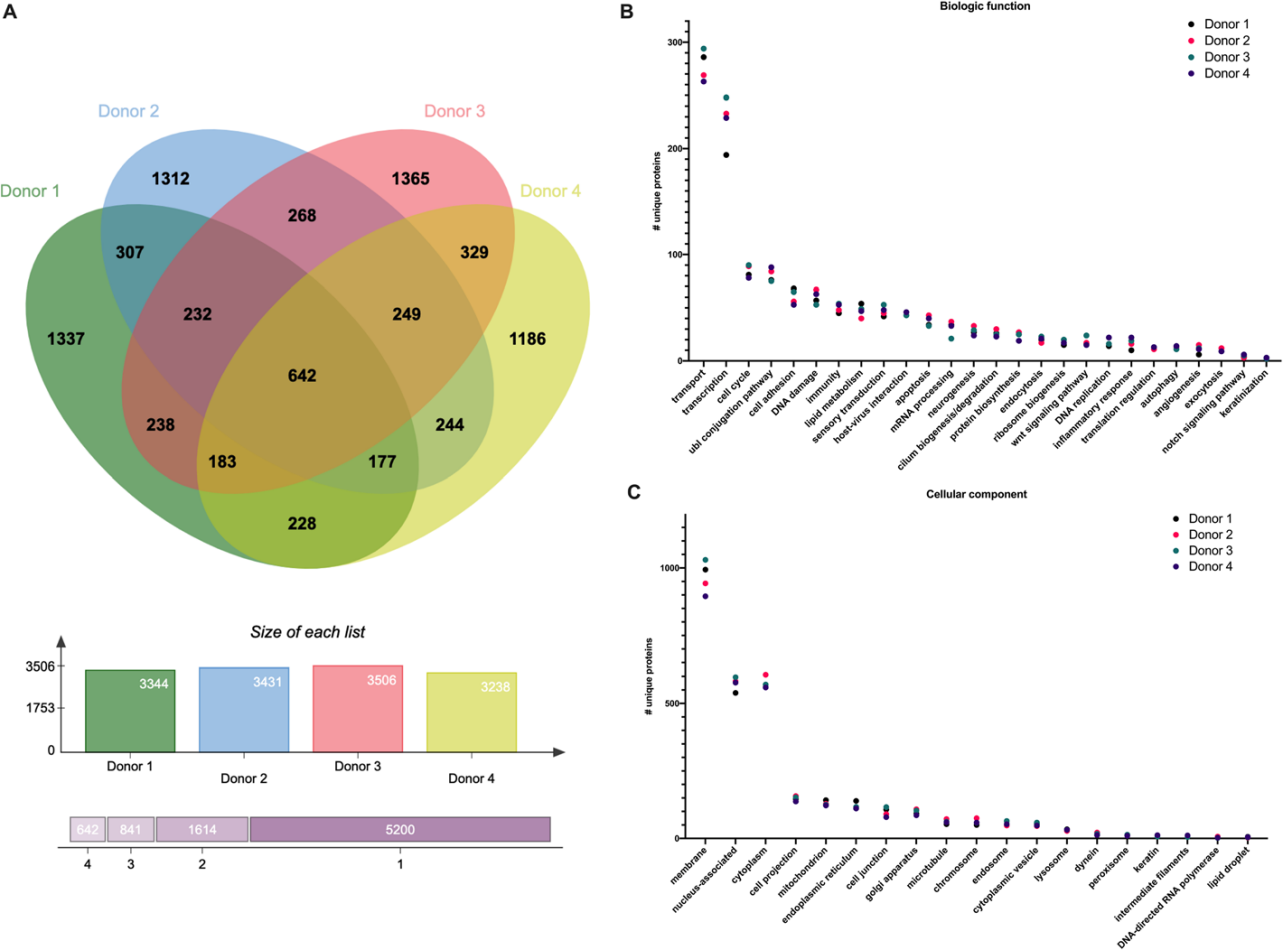
BM-MSC EVs contain diverse protein cargo relevant to a wide variety of biologic functions. **A** Venn diagram across all 4 healthy human donors. **B** Biologic functions and **C** cellular components of BM-MSC EVs across all 4 donors (# of unique proteins per category)

The four donors each contained more than 3000 unique proteins identified within their EV cargo (Fig. 1A). More than 600 of these proteins were in common among all four donors (Fig. 1A). In terms of biologic function, the proteins among all donors had similar numbers of unique proteins among each functional category (Fig. 1B). The most common functional categories were proteins involved in transport (especially transport of ions and other proteins), followed by transcription, cell cycle, ubiquitin conjugation pathways, cell adhesion, deoxyribonucleic acid (DNA) damage, immunity, lipid metabolism, sensory transduction, host-virus interaction, apoptosis, messenger ribonucleic acid (mRNA) processing, neurogenesis, cilium biogenesis/degradation, protein biosynthesis, endocytosis, ribosome biogenesis, Wnt signaling, DNA replication, inflammatory response, translation regulation, autophagy, angiogenesis, exocytosis, notch signaling, and keratinization (Fig. 1B). In terms of the cellular component with which the proteins were associated, the most common were proteins associated with the cell membrane, followed by the nucleus, cytoplasm, cell projections, mitochondrion, endoplasmic reticulum, cell junctions, golgi apparatus, microtubules, chromosomes, endosomes, cytoplasmic vesicles, lysosomes, dynein, peroxisomes, keratin, intermediate filaments, DNA-directed RNA polymerase, and lipid droplets (Fig. 1C).

**Fig. 2:**
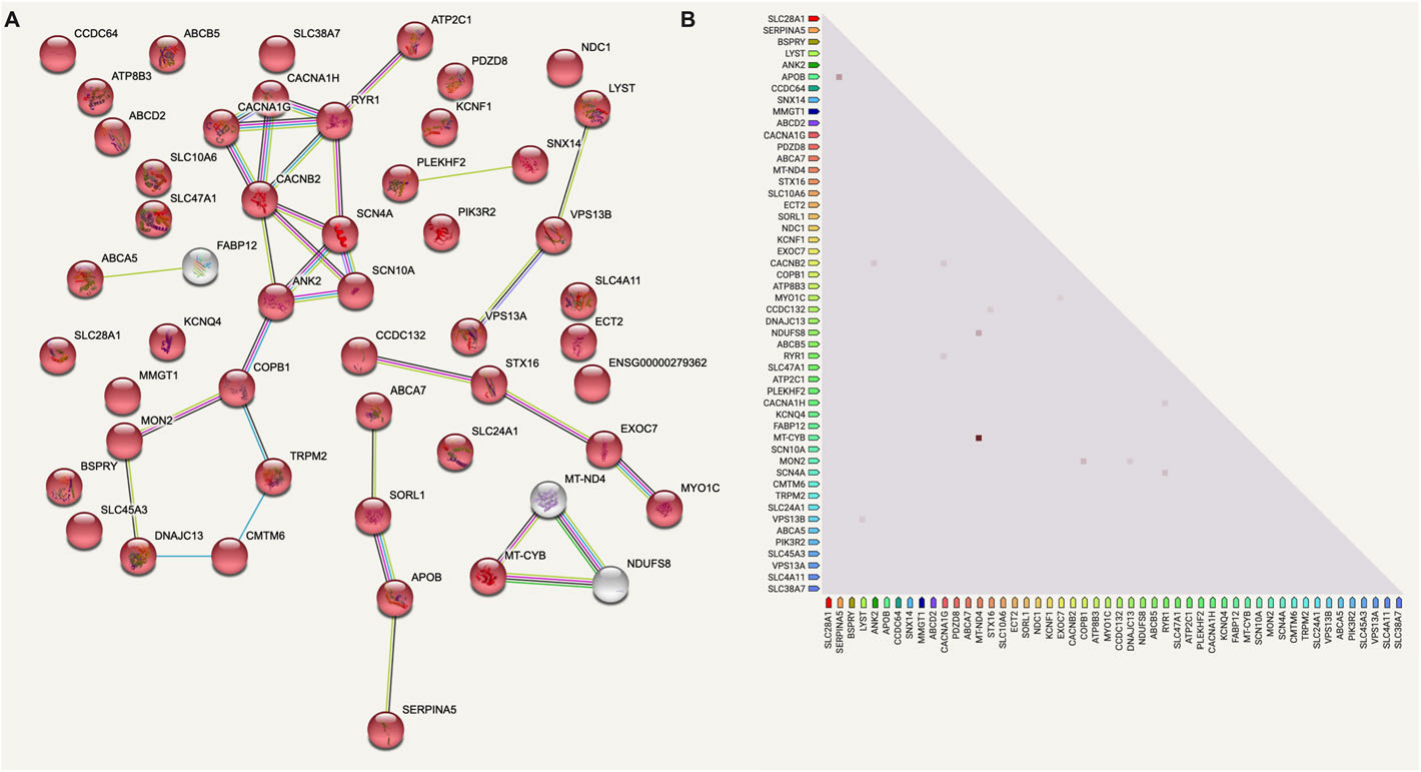
BM-MSC EVs contain important proteins involved in the transport of biologically active proteins and ions. **A** Proteomic network involving proteins in all four donors classified as protein or ion transporters. **B** Coexpression map based on STRING database aggregation of experimental data in which proteins are known to be expressed together

Using the STRING consortium database, we visualized the structural and functional networks among the common proteins involved in transport (Fig. 2A). Central among the network were calcium transport-related proteins, such as the Voltage-dependent T-type calcium channel subunits alpha-1G (CACNA1G) and alpha-1H (CACNA1H) (Fig. 2A), which mediate the entry of calcium ions into cells and is involved in cell motility, cell division, and gene expression [9, 10]. Closely related in this hub was the voltage-dependent L-type calcium channel subunit beta-2 (CACNB2) (Fig. 2A), which increases the peak calcium current across cell membranes [11]. Ryanodine receptor 1 (RYR1), another calcium channel that is also expressed in epidermal keratinocytes and associated with keratinocyte differentiation and epidermal permeability barrier homeostasis [12], was detected in all donor EVs (Fig. 2A). Calcium-transporting ATPase type 2C member 1 (ATP2C1), a magnesium-dependent enzyme that is critical in calcium homeostasis and keratinocyte adhesion, was functionally connected to the aforementioned proteins (Fig. 2A) [13]. Several sodium-related channels were discovered in EVs. The sodium channel proteins type 4 subunit alpha (SCN4A) and type 10 subunit alpha (SCN10A) were present (Fig. 2A) [14]. Transient receptor potential cation channel subfamily M member 2 (TRPM2), a voltage-independent cation channel mediating both sodium and calcium influx, was detected (Fig. 2A) [15]. The detected transport proteins also included endosomal trafficking-related proteins, such as DnaJ homolog subfamily C member 13 (DNAJC13) [16, 17] (Fig. 2A), which is involved in membrane trafficking through early endosomes and implicated in recycling epidermal growth factor receptor. Coatomer subunit beta (COPB1) [18, 19] (Fig. 2A) is a cytosolic protein that associates with vesicles from the Golgi apparatus and mediating protein transport from the endoplasmic reticulum. BM-MSC EV cargo contained proteins involved in electron transport that have been shown to be co-expressed together in independent experiments of human cells (Fig. 2B) (STRING database analytics). NADH-ubiquinone oxidoreductase chain 4 (MT-ND4) [20], a core subunit of the mitochondrial membrane respiratory chain NADH dehydrogenase, which plays a critical role in the electron transport chain, was co-expressed with cytochrome b (MT-CYB) [21] (Fig. 2B), which is a component of the ubiquinol-cytochrome c reductase complex, also a critical component of the respiratory chain, ultimately contributing to the synthesis of ATP needed for cellular processes. Overall, the donors all shared BM-MSC EV cargo proteins essential to ion, protein, and electron transport.

**Fig. 3:**
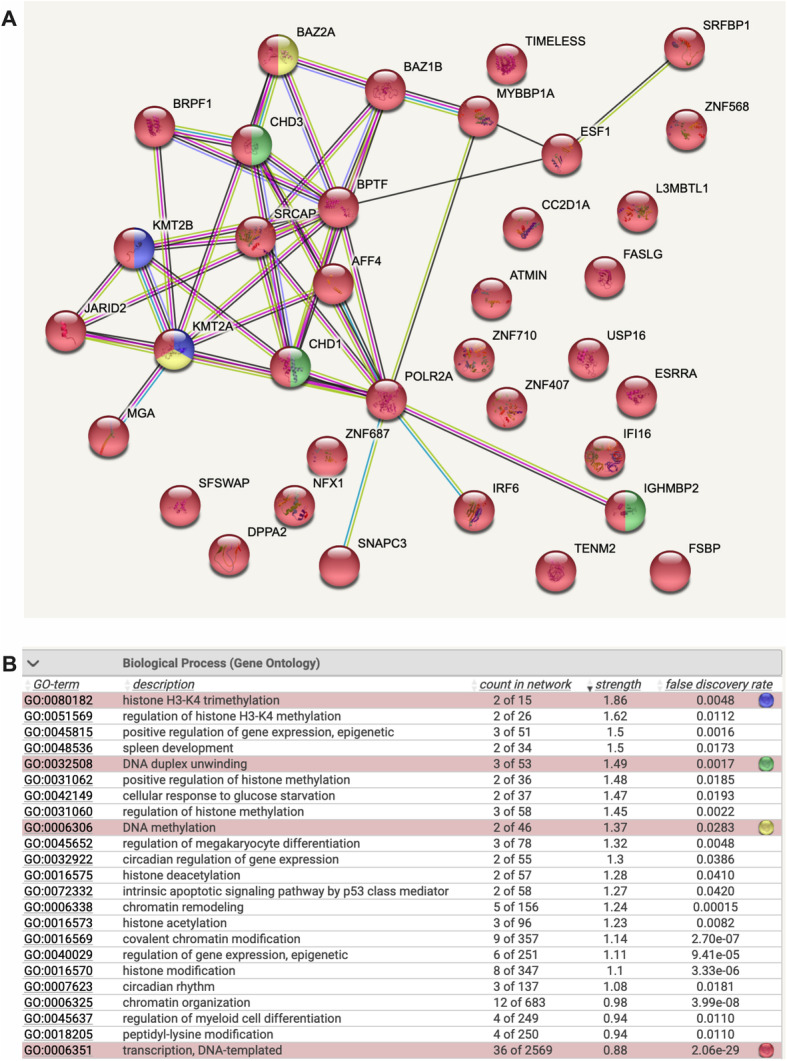
BM-MSC EVs contain cargo important for transcriptional regulation. **A** Proteomic network involving proteins in all four donors classified as transcriptional regulators. **B** List of gene ontology identifiers and corresponding color key as indicated (blue = histone H3-K4 trimethylation; green = DNA duplex unwinding; yellow = DNA methylation; red = transcription, DNA-templated)

All donor BM-MSC EV cargo contained important transcriptional regulators. DNA-directed RNA polymerase II subunit RPB1 (POLR2A) [22] was central in the network hub (Fig. 3A). POLR2A is the largest component of RNA polymerase II and catalyzes the transcription of DNA into RNA. AF4/FMR2 family member 4 is a component of the super elongation complex (SEC), which increases the catalytic rate of RNA polymerase II transcription (Fig. 3A) [23]. Epigenetic modulators, such as histone-lysine N-methyltransferases 2A and 2B (KMT2A and KMT2B) (Fig. 3A) were present in all donor EVs [24]. Also present were chromodomain-helicase-DNA-binding proteins 1 and 3 (CHD1 and CHD3) (Fig. 3A) [25]. CHD1 is an ATP-dependent chromatin-remodeling protein associated with the histone acetylation (HAT) complex regulating RNA polymerase transcription; CHD3 is a component of the histone deacetylase NuRD complex involved in epigenetic regulation. The helicase, SRCAP, belongs to the SNF2/RAD54 helicase family and mediates ATP-dependent histone modification [26] (Fig. 3A). Jumonji (JARID2) (Fig. 3A) is a regulator of histone methyltransferase by promoting recruitment of histone methyltransferase complexes to their target genes [27]. Bromodomain adjacent to zinc finger domain proteins 2A and 1B (BAZ2A and BAZ1B) (Fig. 3A) were detected. BAZ2A is an essential component of the nucleolar remodeling complex (NoRC) [28]. BAZ1B is an atypical tyrosine-protein kinase that plays a central role in chromatin remodeling as a component of the WICH complex, which mobilizes nucleosomes and reconfigures chromatin [29]. The enriched functions of the proteins detected in all donors were concentrated in histone H3-K4 trimethylation, epigenetic gene regulation, DNA duplex unwinding, and DNA methylation among others (Fig. 3B).

**Fig. 4:**
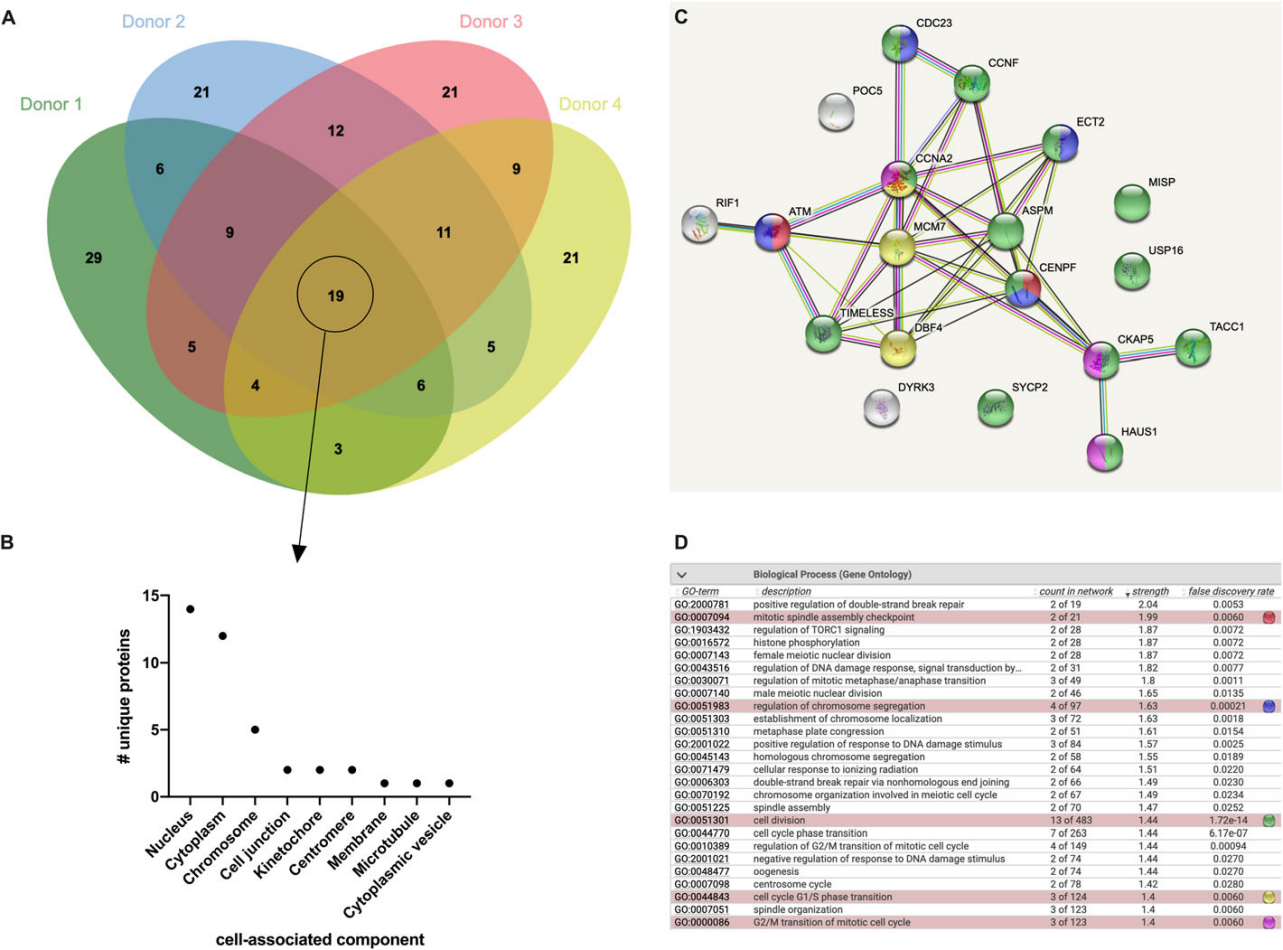
BM-MSC EVs contain cell cycle-related proteins. **A** Venn diagram of cell cycle-related proteins across donors. **B** Cell-associated components of the cell cycle-related proteins. **C** Proteomic network of cell cycle-related proteins. **D** List of gene ontology identifiers and corresponding color key as indicated (red = mitotic spindle assembly checkpoint; blue = regulation of chromosome segregation; green = cell division; yellow = cell cycle G1/S phase transition; purple = G2/M transition of mitotic cell cycle)

There were 19 cell cycle-related proteins that were detected in all four donors (Fig. 4A). Most of these proteins were associated with functions in the nucleus (Fig. 4B). MCM7 (Fig. 4C) is a DNA replication licensing factor which is a replicative helicase essential for DNA replication [30]. Timeless (Fig. 4C) plays an important role in DNA replication via maintenance of replication fork and genome stability [31, 32]. Protein DBF4 (Fig. 4C) plays a central role in DNA replication and cell proliferation. Serine-protein kinase ATM (Fig. 4C) activates checkpoint signaling upon DNA damage [33, 34]. RIF1 (Fig. 4C) is a telomere-associated protein that plays a role in double-strand DNA breaks and promotes non-homologous end joining-mediated repair [35–37]. Specific cyclins were conserved among the donors’ BM-MSC EVs. Cyclin-A2 (CCNA2) (Fig. 4C) controls G1/S and G2/M transition phases in the cell cycle and complexes with cyclin-dependent protein kinases CDK1 and CDK2 [38]. Cyclin-F (CCNF) (Fig. 4C) is a substrate recognition component of the SKP1-CUL-F-box protein E3 ubiquitin-protein ligase complex that mediates proteasomal degradation to inhibit centrosome duplication. Cell division cycle protein 23 homolog (CDC23) (Fig. 4C) is a component of the anaphase promoting complex/cyclosome (APC/C), which is a cell cycle-regulated E3 ubiquitin ligase that controls cell cycle progression [39]. Centromere protein F (CENPF) is required for kinetochore functions and segregation of chromosomes in mitosis [40]. Abnormal spindle-like microcephaly-associated protein (ASPM) (Fig. 4C) is involved in the regulation of the mitotic spindle [41]. ECT2 (Fig. 4C) is a guanine nucleotide exchange factor that acts on Rho family members and plays roles in signal transduction and cytokinesis [42]. Cytoskeleton-associated protein 5 (CKAP5) (Fig. 4C) binds to microtubules and regulates the organization of microtubules [43].

**Fig. 5:**
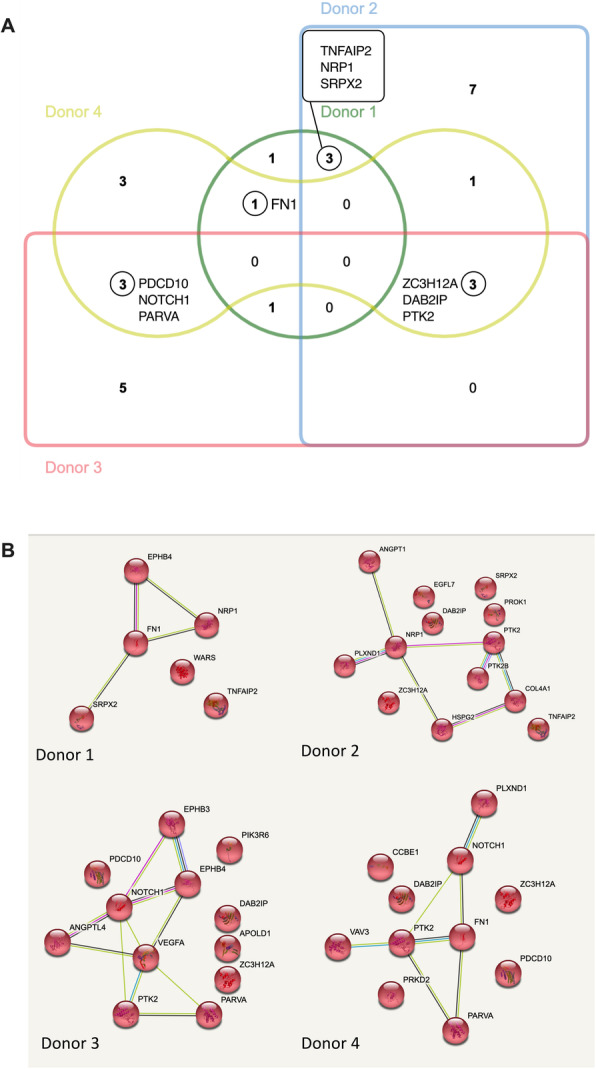
BM-MSC EVs contain angiogenesis-related cargo. **A** Venn diagram of angiogenesis-related proteins across donors. **B** Individual proteomic network maps of angiogenesis-related proteins across donors

We hypothesized that BM-MSC EVs would contain angiogenesis-related cargo. Donor 1 and donor 2 shared three angiogenesis-related proteins in common: Neuropilin-1 (NRP1) [44–50], tumor necrosis factor alpha-induced protein 2 (TNFAIP2) [51], and Sushi repeat-containing protein (SRPX2) [52] (Fig. 5A), which have all been demonstrated to be modulators of angiogenesis. Donors 3 and 4 shared three angiogenesis-related proteins in common (Fig. 5A): NOTCH1 functions as a receptor for membrane-bound ligands Jagged1, Jagged2, and Delta1 to regulate cell-fate and modulate angiogenesis [53–55]; programmed cell death protein 10 (PDCD10) promotes cell proliferation and modulates apoptosis to regulated angiogenesis [56]; PARVA plays a role in sprouting angiogenesis and is required for normal adhesion of vascular smooth muscle cells during blood vessel development [57]. Donors 1 and 4 both contained fibronectin 1 (FN1) (Fig. 5A), which has been implicated in the modulation of angiogenesis [58]. Donors 2, 3, and 4 contained three proteins in common (Fig. 5A): disabled homolog 2-interacting protein (DAB2IP) is a scaffold protein that regulates cell migration and angiogenesis [59]; focal adhesion kinase (PTK2) is a tyrosine kinase that regulates cell migration, adhesion, and endothelial cell spreading [60]; endoribonuclease ZC3H12A is involved in mRNA decay and regulates many biologic processes, including angiogenesis [61]. Overall, donors showed wide variability in angiogenesis-related proteins, with some conservation of proteins among selected donors.

**Fig. 6:**
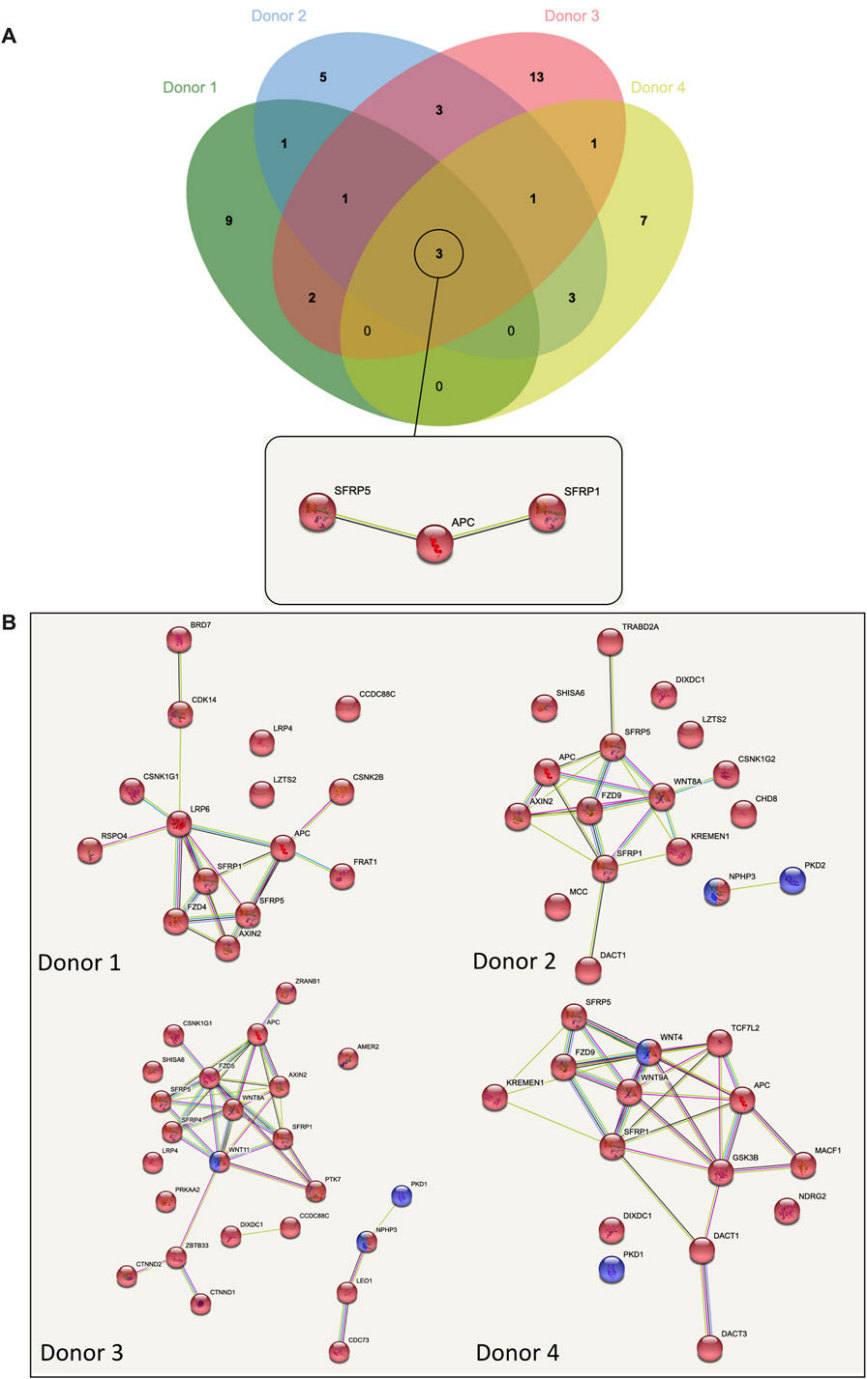
BM-MSC EVs contain Wnt signaling-related proteins. **A** Venn diagram of Wnt signaling-related proteins across donors. **B** Individual proteomic network maps of Wnt signaling-related proteins across donors

Wnt signaling activity has been demonstrated to be important in cutaneous wound healing. We hypothesized that BM-MSC EVs would contain Wnt signaling modulators. All donors’ BM-MSC EVs contained the tumor adenomatous polyposis coli (APC) protein (Fig. 6A), which promotes rapid degradation of beta-catenin and consequently regulates Wnt signaling activity [62]. Secreted frizzled-related proteins 1 and 5 (SFRP1 and SFRP5) (Fig. 6A) were also present in all donor BM-MSC EVs. SFRPs function as modulators of Wnt signaling via direct interactions with Wnt ligands in the extracellular environment [63]. Depending on the type of Wnt ligands they bind, SFRPs can induce or inhibit canonical Wnt signaling, which may have differing temporal effects on processes such as angiogenesis and fibrosis during cutaneous wound healing [64, 65]. Various Wnt ligands were expressed in some, but not all, donors. For example, donors 2 and 3 EVs contained WNT8A, while donor 3 contained WNT11 and donor 4 contained WNT4 and WNT9A (Fig. 6B). We found that Wnt receptors were present in BM-MSC EVs. Frizzled (Fz) receptors were found in all donors (Fig. 6B). Low-density lipoprotein receptor-related protein 6 (LRP6) was present in donor 1 EVs, while LRP4 was present in donors 1 and 3 (Fig. 6B). AXIN2, present in donors 1, 2, and 3 (Fig. 6B), is a component of Wnt signaling that is involved in beta-catenin degradation [66]. Overall, the BM-MSC EVs exhibit both conserved cargo and significant variation that may alter the balance of Wnt signaling.

**Fig. 7:**
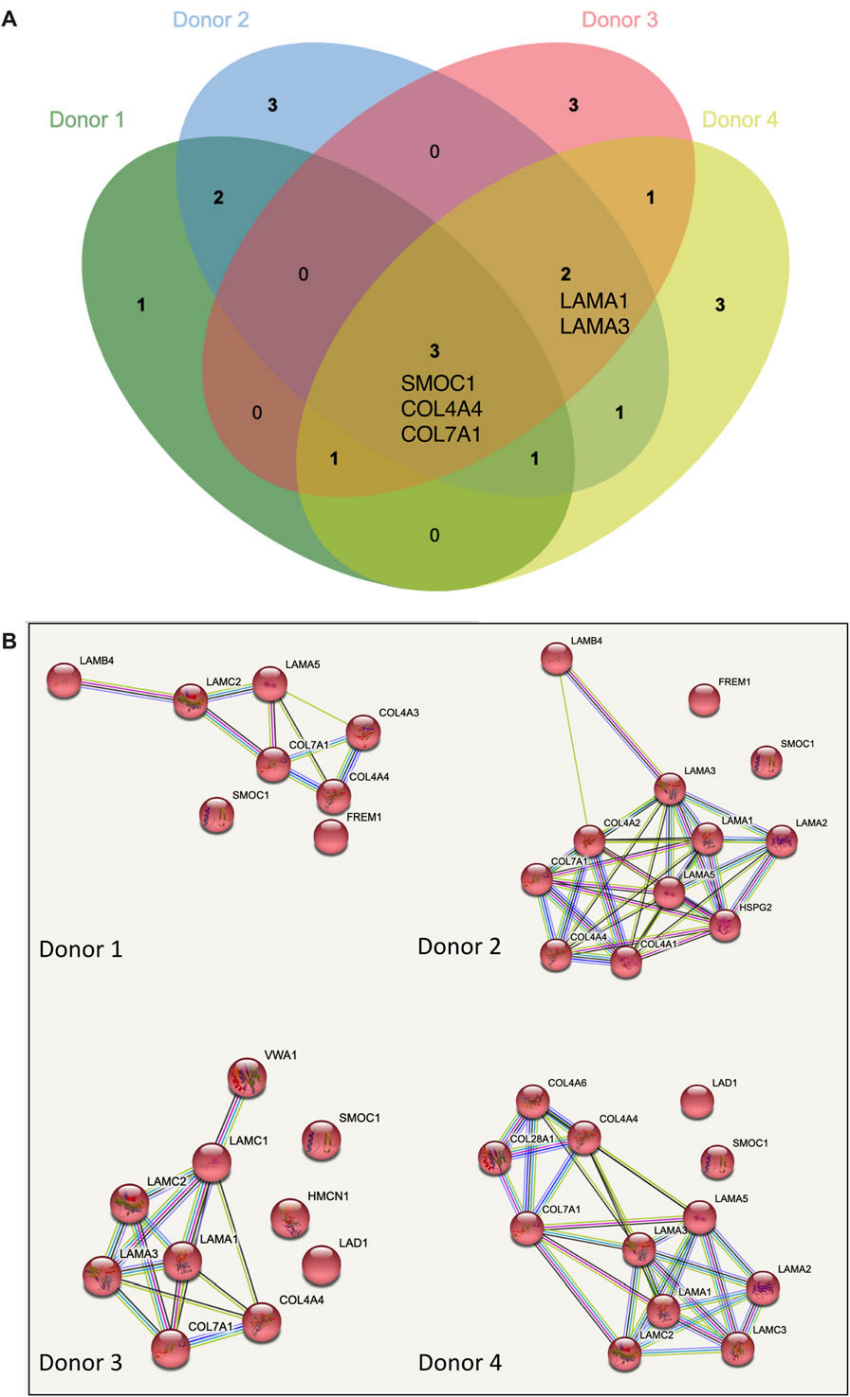
BM-MSC EVs contain basement membrane proteins. **A** Venn diagram of basement membrane-related proteins across donors. **B** Individual proteomic network maps of basement membrane-related proteins across donors

Given our previous findings, we hypothesized that BM-MSC EVs would contain important basement membrane proteins. All donors’ EVs contained multiple subunits of collagen IV and VII (Fig. 7A, B) [67–69], which are critical in the formation of the skin basement membrane. Donors 2, 3, and 4 contained laminin subunits A1 and A3 (LAMA1 and LAMA3) (Fig. 7A, B), which are crucial in the formation of the basement membrane. Thus, BM-MSC EVs could carry cargo proteins to healing wounds in both damaged skin and in patients with genetic deficiencies.

## Discussion

Our study finds that healthy donors of BM-MSC EVs contain important similarities and differences that should be considered in the development of EVs as therapeutics. BM-MSC EVs carry functional cargo important for a wide variety of biologic processes, including transportation of proteins and ions, transcription, cell cycle, and epigenetic processes, but this list is not exhaustive. With relevance to cutaneous wound healing, BM-MSC EVs could play a key role in the promotion of repair and regeneration via its modulation of cell proliferation and angiogenesis and critical signaling pathways, such as Wnt signaling. Furthermore, replenishment of basement membrane proteins is critical to repair and regeneration. An important future avenue of investigation would involve comparing BM-MSC EVs from healthy donors and patients with various diseases (such as chronic wound healing or diabetes); however, we recognize the ethical challenges in obtaining such bone marrow samples in patients at risk for potential complications related to invasive procedures. Additionally, it would be important for screening to understand if there are key, circulating biomarkers in the blood that could predict the relevant cargo that might be contained in a donor’s BM-MSC EVs, before isolating the bone marrow. Ideally, some of the key protein cargo from the BM-MSCs identified as useful in the promotion of cutaneous regeneration would be available for detection in the circulation, allowing for a more optimal screening strategy. One limitation of our study is that we only assessed four healthy donors; further studies on a larger number of donors, across different age groups, in independent institutions are needed to help validate cargo signatures in BM-MSC EVs. Furthermore, efforts to correlate proteomic (and genomic) signatures to functional outcomes (in in vitro potency assays and clinical trials) are warranted. Given the importance of stem cells in the development of therapeutics, BM-MSC EVs may play an important role in translational therapeutic development in cutaneous wound repair and regeneration.

## Conclusion

BM-MSCs contain important protein cargo that makes them significant candidates for endogenous contributors and therapeutic candidates for cutaneous wound repair and regeneration. Donor screening for clinical trials is warranted for ultimate application to examine the effects of BM-MSCs on recipient wound healing in a variety of disease conditions.

## Data Availability

JM, LRM, and EB will store raw data and make available upon request.

## Abbreviations

ASPM: Abnormal spindle-like microcephaly-associated protein
ANC: Acetonitrile
APC: Adenomatous polyposis coli
APC/C: Anaphase promoting complex/cyclosome
BM-MSCs: Bone marrow mesenchymal stem cells
BAZ2A and BAZ1B: Bromodomain adjacent to zinc finger domain proteins 2A and 1B
ATP2C1: Calcium-transporting ATPase type 2C member 1
CENPF: Centromere protein F
CHD1 and CHD3: Chromodomain-helicase-DNA-binding proteins 1 and 3
CMV: Cytomegalovirus
COPB1: Coatomer subunit beta
CCNA2: Cyclin-A2
CCNF: Cyclin-F
MT-CYB: Cytochrome b
CKAP5: Cytoskeleton-associated protein 5
DAB2IP: Disabled homolog 2-interacting protein
DTT: Dithiothreitol
DNA: Deoxyribonucleic acid
POLR2A: DNA-directed RNA polymerase II subunit RPB1
DNAJC13: DnaJ homolog subfamily C member 13
EVs: Extracellular vesicles
FDR: False discover rate
FN1: Fibronectin 1
GMP: Good Manufacturing Practices
HBc: Hepatitis B core antigen
HBsAg: Hepatitis B surface antigen
HCV: Hepatitis C virus
HESI: Heated electrospray ionization source
HAT: Histone acetylation
KMT2A and KMT2B: Histone-lysine N-methyltransferases 2A and 2B
HIV: Human immunodeficiency virus
HPLC: High-performance liquid chromatography
HSA: Human serum albumin
HTLV: Human T-lymphotrophic virus
JARID2: Jumonji
LAMA1 and LAMA3: Laminin subunits A1 and A3
LC-MS: Liquid chromatography/mass spectrometry
LSM: Lymphocyte separation media
MNCs: Mononuclear cells
mRNA: Messenger ribonucleic acid
MT-ND4: NADH-ubiquinone oxidoreductase chain 4
NRP1: Neuropilin-1
NoRC: Nucleolar remodeling complex
PBS: Phosphate-buffered saline
PDCD10: Programmed cell death protein 10
PTK2: Focal adhesion kinase
RPR: Rapid plasma reagin
RYR1: Ryanodine receptor 1
SDS: Sodium dodecyl sulfate
SFRP1 and SFRP5: Secreted frizzled-related proteins 1 and 5
SCN4A: Sodium channel proteins type 4 subunit alpha
SRPX2: Sushi repeat-containing protein
TNFAIP2: Tumor necrosis factor alpha-induced protein 2
TRPM2: Transient receptor potential cation channel subfamily M member 2
CACNB2: Voltage-dependent L-type calcium channel subunit beta-2
CACNA1G: Voltage-dependent T-type calcium channel subunits alpha-1G
Wnt: Wingless-related integration site
WNV: West Nile virus
